# Supplementary material for: An ethylene biosynthesis enzyme controls quantitative variation in maize ear length and kernel yield
Source: Nat Commun. 2021 Oct 5;12:5832. doi: 10.1038/s41467-021-26123-z (PMC8492687; doi:10.1038/s41467-021-26123-z)
Supplement: Supplementary file 1 — Supplementary Information [file 41467_2021_26123_MOESM1_ESM.pdf]

Supplementary information

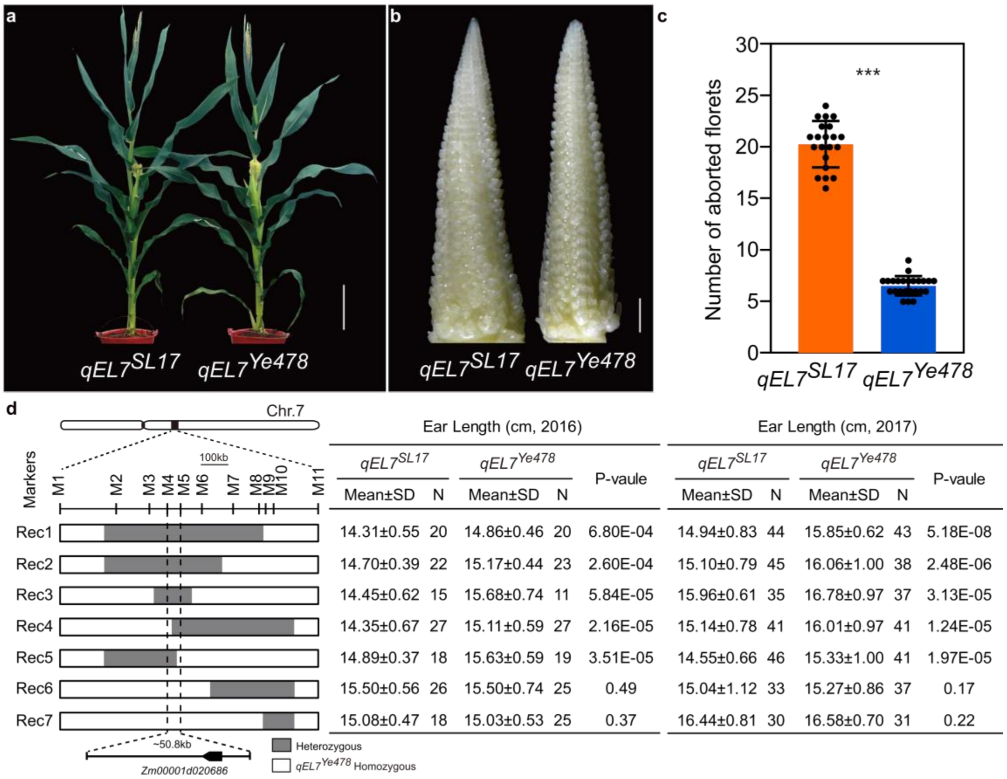

	Ear Length (cm, 2016)					Ear Length (cm, 2017)				
	qEL7^{SL17}		*qEL7*^{Ye478}		P-vaule	*qEL7*^{SL17}		*qEL7*^{Ye478}		P-vaule
	Mean±SD	N	Mean±SD	N		Mean±SD	N	Mean±SD	N	
Rec1	14.31±0.55	20	14.86±0.46	20	6.80E-04	14.94±0.83	44	15.85±0.62	43	5.18E-08
Rec2	14.70±0.39	22	15.17±0.44	23	2.60E-04	15.10±0.79	45	16.06±1.00	38	2.48E-06
Rec3	14.45±0.62	15	15.68±0.74	11	5.84E-05	15.96±0.61	35	16.78±0.97	37	3.13E-05
Rec4	14.35±0.67	27	15.11±0.59	27	2.16E-05	15.14±0.78	41	16.01±0.97	41	1.24E-05
Rec5	14.89±0.37	18	15.63±0.59	19	3.51E-05	14.55±0.66	46	15.33±1.00	41	1.97E-05
Rec6	15.50±0.56	26	15.50±0.74	25	0.49	15.04±1.12	33	15.27±0.86	37	0.17
Rec7	15.08±0.47	18	15.03±0.53	25	0.37	16.44±0.81	30	16.58±0.70	31	0.22

Supplementary Figure 1. Plant and ear phenotypes of two parental lines and recombinant lines.

a-b, *qEL7*^{Ye478} has a similar plant architecture (**a**, scale bar = 20 cm) and less florets per row in developing ear inflorescence (**b**, scale bar = 2 mm) comparing with *qEL7*^{SL17}. **c**, The number of aborted florets of the immature ears in *qEL7*^{SL17} (orange bar) and *qEL7*^{Ye478} (blue bar); $p = 4.44 \times 10^{-29}$, $n = 21$ and 24 ears, respectively. **d**, Ear length of seven recombinant lines were measured using a randomized block design at Wuhan, in 2016 and 2017 spring. Narrow down *qEL7* to a ~50.8 kb physical interval with only one predicted gene, *ZmACO2* (*Zm00001d020686*), in B73 RefGen v4. Data are shown as the mean ± SD. *** p-value ≤ 0.001, from a two-tailed, two-sample t-test.

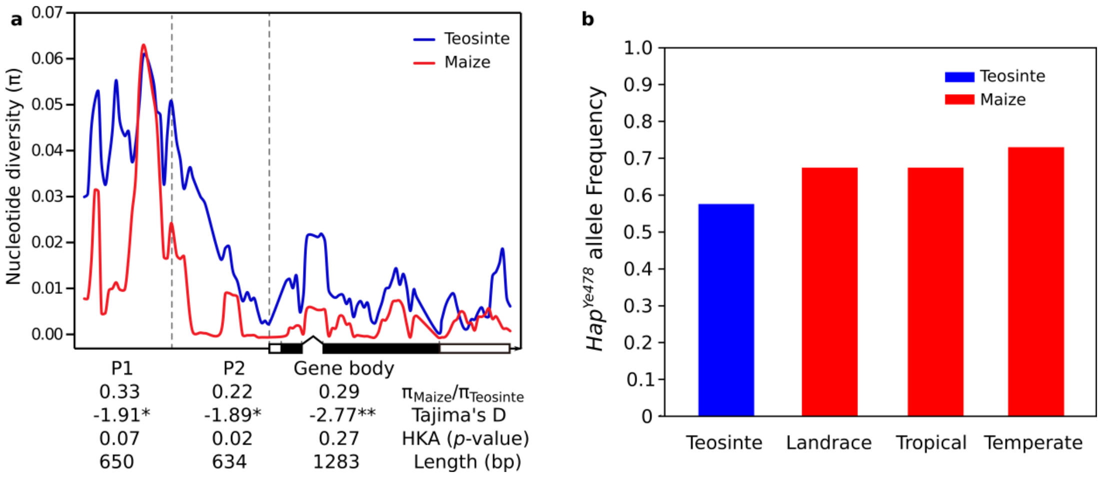

Supplementary Figure 2. The evidence of selection in *ZmACO2* during maize domestication and improvement.

a: Nucleotide diversities (π) within *ZmACO2* promoter and gene body for maize (red line) and teosinte (blue line); the promoter region was divided into two parts (P1 and P2); P2 contains the candidate causal mutations; the white boxes in gene body represents UTR region, black box represents exon, arrowhead represents gene direction; the detail statistics data of $\pi_{maize}/\pi_{teoisnte}$, Tajima's D, HKA test *p*-values, length were listed under the chart; * *p*-value < 0.05, ** *p*-value < 0.01. **b**: Allele frequencies of Hap^{Ye478} in teosinte (blue bar) and maize (red bar, including landrace, tropical and temperate maize inbred lines).

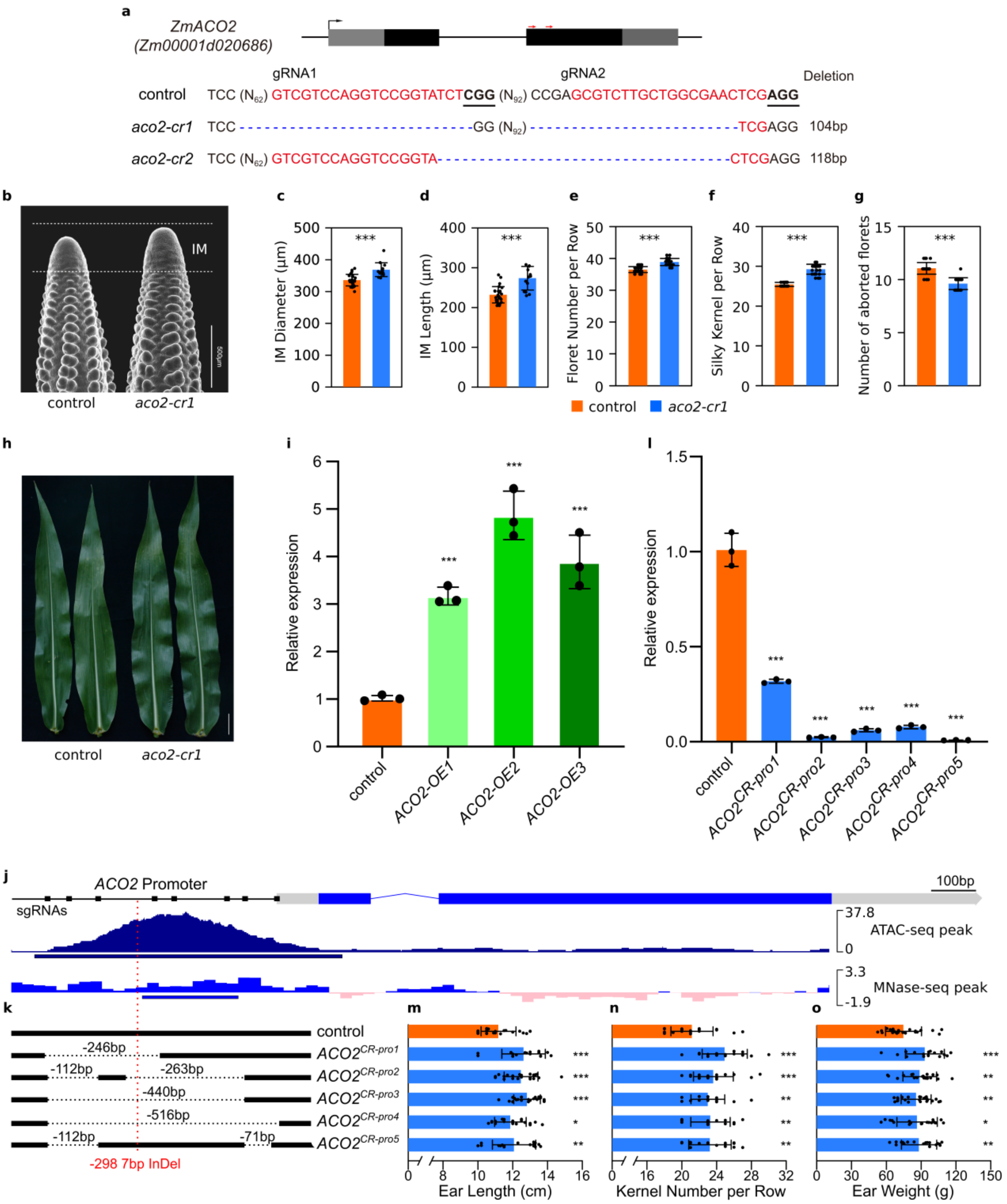

Supplementary Figure 3. Validation of *ZmACO2* function by knockout, overexpression and promoter CRISPR.

a, Gene models, gRNA target sites (red arrows), and *ZmACO2* CRISPR-Cas9-induced knockout alleles. **b**, Scanning electron microscope image of 5-mm ear tip from control and *aco2-cr1*, scale bar = 500 μm; ears from five plants of each genotype were imaged by scanning electron microscope. **c-g**, Phenotypes of IM length (**c**, p = 9.54×10^{-6}) and diameters (**d**, $p = 6.48\times10^{-6}$), floret number per row (**e**, $p = 1.75\times10^{-13}$), silky kernels per row (**f**, $p = 1.27\times10^{-24}$) and number of aborted florets (**g**, p = 1.56×10^{-15}) of control and *aco2-cr1*; **c-d**: 5-mm ear stage, n = 26 and 14 for control and *aco2-cr1*, respectively; **e-g**: immature ears before pollination, n = 34 and 31 for

control and *aco2-cr1*, respectively; orange bar, control; blue bars, *aco2-cr1*. **h**, Photos of mature leaves in control and *aco2-cr1*; scale bar = 5 cm. **i**, *ZmACO2* expression level in ear primordia of three overexpression lines ($p = 2.31 \times 10^{-5}$, 1.01×10^{-4} and 4.67×10^{-4} respectively). **j**, The distribution of sgRNA target sites cover 520 bp non-repetitive promoter regions of *ZmACO2*; black box, sgRNAs; blue boxes, exons; grey box, UTR region. The accessible chromatin regions of the *ZmACO2* promoter in developing ear primordia are detected by ATAC-seq[22] and MNase-seq[23]. Dark blue peaks, ATAC-seq peaks; dark blue boxes, open chromatin regions defined by ATAC-seq peaks; light-blue peaks, MNase-seq peaks; light-blue boxes, MNase hypersensitive sites defined by MNase-seq peaks; vertical red dashed lines indicate the possible causal variant (7 bp InDel at -298 bp from TSS) of *qEL7*. **k**, Five promoter-edited alleles of *ZmACO2*. The deletion (-) base pairs are indicated by numbers. **l**, The normalized relative expression of *ZmACO2* of homozygous promoter edited alleles were lower than wild type controls measured in 5-mm ear primordia ($p = 8.71 \times 10^{-5}$, 2.08×10^{-5}, 2.47×10^{-5}, 2.67×10^{-5} and 1.96×10^{-4} respectively). **m-o**, Phenotypes of ear length (**m**, $p = 1.48 \times 10^{-4}$, 4.38×10^{-5}, 7.57×10^{-8}, 0.016 and 0.0073 respectively), kernel number per row (**n**, $p = 2.17 \times 10^{-5}$, 8.81×10^{-4}, 0.0065, 0.0039 and 0.0058 respectively) and ear weight (**o**, $p = 5.41 \times 10^{-4}$, 0.0023, 0.0059, 0.013 and 0.0042 respectively) in *ZmACO2* promoter-edited alleles and sibling controls. Orange bar, control, $n = 23$ ears; blue bars, promoter-edited alleles, $n = 16$, 21, 23, 17 and 18 ears, respectively. For **i** and **l**, expression level was measured by qRT-PCR with three biological replicates and three technical replicates. Relative expression level was calculated by the $2^{-\Delta Ct}$ method with maize *Actin* gene (*Zm00001d010159*) as an internal control; approximately 20 ears were used in each biological replicate. For **c-g, i, l** and **m-o**, data are presented as means ± SD. * p-value ≤ 0.05, ** p-value ≤ 0.01, *** p-value ≤ 0.001, from a two-tailed, two-sample t-test. n is the number of ears examined in **c-g** and **m-o**.

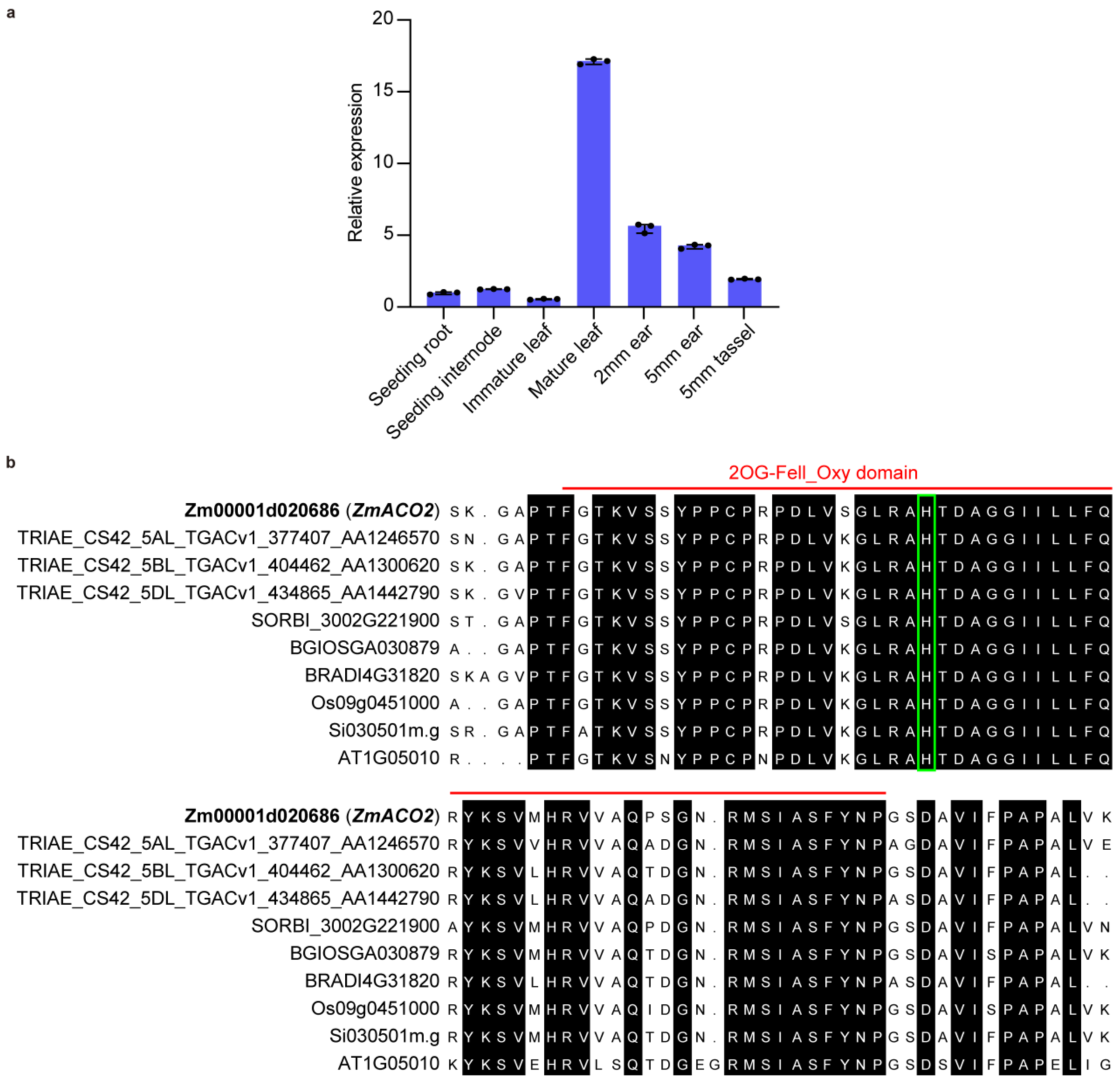

Supplementary Figure 4. Expression pattern of *ZmACO2* and characteristics of ZmACO2 protein.

a, Relative expression level of *ZmACO2* in seeding root, seeding internode, immature leaf, mature leaf, 2 mm and 5 mm ear, and 5mm tassel. Expression level was measured by qRT-PCR with three biological replicates and three technical replicates; samples from 10-20 plants were used in each biological replicate. Relative expression level was calculated by the $2^{-\Delta Ct}$ method with maize *Actin* gene (*Zm00001d010159*) as an internal control. Data are presented as means ± SD. **b**, Alignment of the Fe^{2+} and 2OG-dependent dioxygenase domain (2OG-FeII_Oxy domain) with *ZmACO2* homologs from diverse species. The red line represents the 2OG-FeII_Oxy domain. The blue rectangle indicates a conserved histidine residue that participates in binding Fe^{2+}.

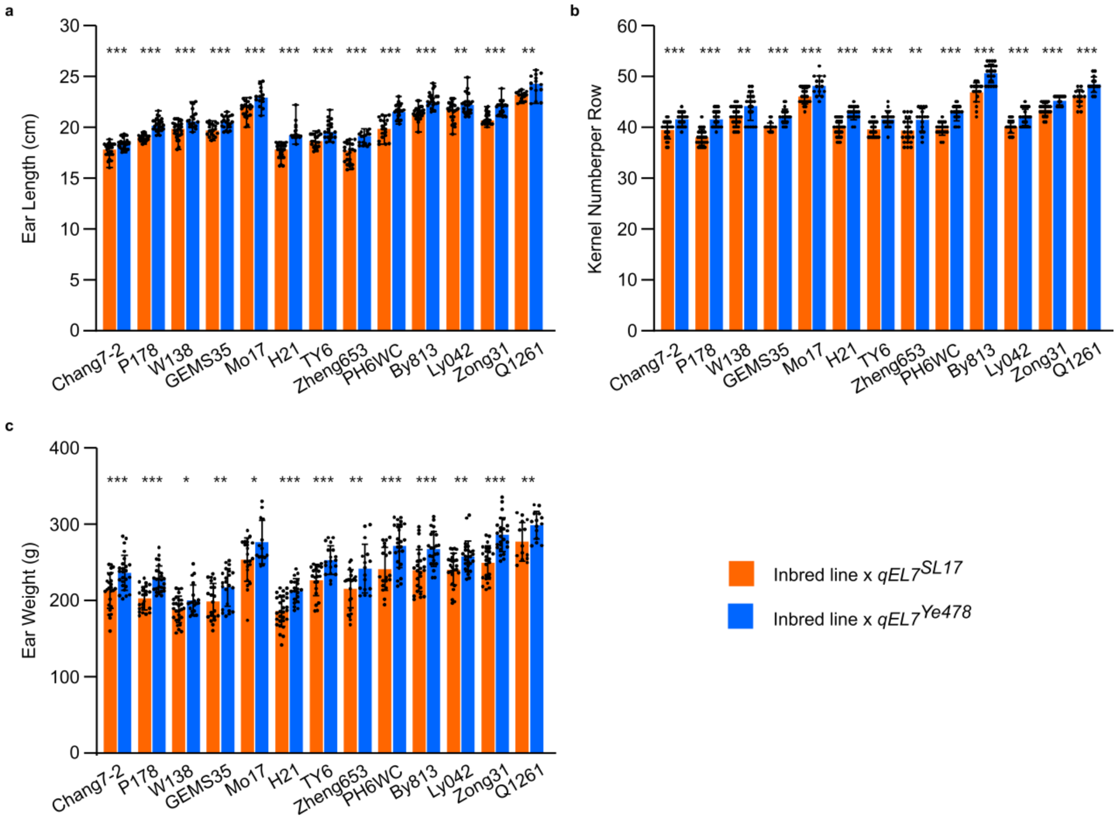

Supplementary Figure 5. The ear-trait phenotypes of hybrids derived from two NILs crossing to a set of inbred lines.

a-c, Performances of hybrids (a-c) in grain ear-traits including ear length (**a**, p = 1.58×10^{-4}, 2.14×10^{-11}, 8.06×10^{-5}, 1.89×10^{-4}, 9.22×10^{-5}, 3.90×10^{-10}, 6.50×10^{-5}, 2.77×10^{-6}, 5.19×10^{-8}, 4.76×10^{-8}, 0.0049, 7.97×10^{-7} and 0.0014 respectively), kernel number per row (**b**, p = 6.73×10^{-6}, 1.96×10^{-10}, 0.0022, 3.27×10^{-7}, 4.22×10^{-4}, 3.06×10^{-9}, 7.31×10^{-5}, 0.0036, 3.72×10^{-9}, 3.83×10^{-9}, 8.57×10^{-6}, 1.16×10^{-7} and 1.11×10^{-4} respectively), and ear weight (**c**, p = 4.08×10^{-4}, 1.14×10^{-6}, 0.015, 0.0075, 0.012, 4.94×10^{-6}, 6.89×10^{-5}, 0.0032, 6.11×10^{-4}, 1.88×10^{-4}, 0.0021, 3.26×10^{-7} and 0.0072 respectively) developed by 13 inbred lines separately crossing to $qEL7^{SL17}$ and $qEL7^{Ye478}$. Orange bar, "inbred line × $qEL7^{SL17}$", n = 25, 23, 27, 21, 23, 29, 21, 23, 19, 25, 27, 24, 14 ears, respectively; blur bar, "inbred line × $qEL7^{Ye478}$". n = 25, 26, 20, 22, 14, 20, 19, 16, 25, 23, 26, 25, 14 ears, respectively. Data are presented as means ± SD. * p-value ≤ 0.05, ** p-value ≤ 0.01, *** p-value ≤ 0.001, from a two-tailed, two-sample t-test.

Supplementary Table 1. Phenotype of agronomic traits of *qEL7*^{SL17} and *qEL7*^{Ye478}

Trait	*qEL7*^{SL17}	*n*	*qEL7*^{Ye478}	*n*	*p*-value
Ear Length (cm)	13.44±0.78	32	14.17±0.42	39	2.03×10^{-6}
Kernel Number per Row	24.25±1.22	32	26.62±0.71	39	1.03×10^{-15}
Kernel Row Number	13.25±0.98	32	13.13±1.00	39	0.30
Ear Diameter (mm)	39.25±1.30	32	39.79±2.08	39	0.10
100-Kernel Weight (g)	29.77±0.90	15	33.72±1.43	14	7.42×10^{-10}
Ear Weight (g)	82.70±14.15	32	103.68±8.05	39	1.90×10^{-11}
IM Length (μm)	294.58±19.54	31	234.39±17.74	31	5.92×10^{-19}
IM Diameter (μm)	342.39±19.63	31	315.58±16.21	31	1.04×10^{-7}
Floret Number per Row	43.86±2.97	21	32.75±1.29	24	1.24×10^{-20}
Silky Kernel per Row	23.57±0.81	21	26.21±0.51	24	9.04×10^{-17}
Days to heading	73.17±0.39	12	74.83±0.72	12	9.39×10^{-07}
Days to silking	76.58±1.31	12	77.00±1.28	12	0.037
Days to pollen	74.17±0.39	12	76.00±0.85	12	3.13×10^{-06}
Plant height (cm)	176.76±4.21	21	177.91±5.09	22	0.21
Ear leaf length (cm)	60.67±4.20	42	61.56±3.48	45	0.14
Ear leaf width (cm)	8.88±0.86	42	8.74±0.71	45	0.20

p-value: two-tailed, two-sample t-test; *n* is the number of plants.

Supplementary Table 2. Significant association variants across the 3544-bp sequenced region around *ZmACO2* after Bonferroni multiple test correction ($p \leq 1.79 \times 10^{-4}$).

Varients	Allele	Position (Relative to the 5'-UTR, bp)	Frequency	*p*-value
SNP-666	A/G	-666	158/66	1.73×10^{-4}
SNP-645	A/G	-645	158/66	1.73×10^{-4}
SNP-643	A/G	-643	158/66	1.73×10^{-4}
SNP-622	G/A	-622	158/66	1.73×10^{-4}
InDel-298	-------/GCCCGAG	-298	158/66	1.73×10^{-4}

Supplementary Table 3. Transcription factor binding motif prediction in the 7bp InDel region (-298) by PlantPan 3.0.

Allele	Sequence[1]	Transcription factors	Position	Strand	Similar Score	Hit Sequence
qEL7^{SL17}	CCGAG GCCCGAG CTGAC	bHLH	6	+	0.75	GCCCGagc
qEL7^{SL17}	CCGAG GCCCGAG CTGAC	bHLH	6	-	0.75	gccCGAGC
qEL7^{SL17}	CCGAG GCCCGAG CTGAC	C2H2	8	-	0.94	ccgAGCTGac
qEL7^{SL17}	CCGAG GCCCGAG CTGAC	TCP	6	+	1	GCCCG
qEL7^{SL17}	CCGAG GCCCGAG CTGAC	TCP	4	+	0.75	AGGCC
qEL7^{SL17}	CCGAG GCCCGAG CTGAC	TCP	4	-	0.75	AGGCC
qEL7^{SL17}	CCGAG GCCCGAG CTGAC	TCP	5	+	1	GGCCC
qEL7^{SL17}	CCGAG GCCCGAG CTGAC	TCP	5	-	1	GGCCC
qEL7^{SL17}	CCGAG GCCCGAG CTGAC	Dehydrin	1	+	0.8	CCGAG
qEL7^{SL17}	CCGAG GCCCGAG CTGAC	Dehydrin	8	+	0.8	CCGAG
qEL7^{Ye478}	CCGAG CTGAC	C2H2	1	-	0.94	ccgAGCTGac

[1] The GCCCGAG is the 7 bp InDel
