# Supplementary material for: An ethylene biosynthesis enzyme controls quantitative variation in maize ear length and kernel yield
Source: Nat Commun. 2021 Oct 5;12:5832. doi: 10.1038/s41467-021-26123-z (PMC8492687; doi:10.1038/s41467-021-26123-z)
Supplement: Supplementary file 3 — Description of Additional Supplementary Files [file 41467_2021_26123_MOESM3_ESM.pdf]

Description of Additional Supplementary Files

File name: Supplementary Data 1

Description: Phenotypes of yield-related traits of recombinant lines.

File name: Supplementary Data 2

Description: The 40 maize inbred lines used for expression analysis.

File name: Supplementary Data 3

Description: The teosinte and maize landrace lines used in the evolution analysis of ZmACO2 region.

File name: Supplementary Data 4

Description: Phenotypic performance of ZmACO2 CRISPR and OE alleles.

File name: Supplementary Data 5

Description: Phenotypic performance of ZmACO2 promoter CRISPR alleles.

File name: Supplementary Data 6

Description: Expression changes of phytohormones biosynthesis and known inflorescence development-related genes in qEL7Ye478 relative to qEL7SL17.

File name: Supplementary Data 7

Description: The yield-related traits performance in diverse hybrid backgrounds.

File name: Supplementary Data 8

Description: Sequences of primers used in this study.
