# Supplementary material for: An ethylene biosynthesis enzyme controls quantitative variation in maize ear length and kernel yield
Source: Nat Commun. 2021 Oct 5;12:5832. doi: 10.1038/s41467-021-26123-z (PMC8492687; doi:10.1038/s41467-021-26123-z)
Supplement: Supplementary file 12 — Reporting Summary [file 41467_2021_26123_MOESM12_ESM.pdf]

nature portfolio

Corresponding author(s): David Jackson

Last updated by author(s): Sep 3, 2021

Reporting Summary

Nature Portfolio wishes to improve the reproducibility of the work that we publish. This form provides structure for consistency and transparency in reporting. For further information on Nature Portfolio policies, see our Editorial Policies and the Editorial Policy Checklist.

Statistics

For all statistical analyses, confirm that the following items are present in the figure legend, table legend, main text, or Methods section.

n/a	Confirmed	
☐	☒	The exact sample size (*n*) for each experimental group/condition, given as a discrete number and unit of measurement
☐	☒	A statement on whether measurements were taken from distinct samples or whether the same sample was measured repeatedly
☐	☒	The statistical test(s) used AND whether they are one- or two-sided<br>*Only common tests should be described solely by name; describe more complex techniques in the Methods section.*
☒	☐	A description of all covariates tested
☒	☐	A description of any assumptions or corrections, such as tests of normality and adjustment for multiple comparisons
☐	☒	A full description of the statistical parameters including central tendency (e.g. means) or other basic estimates (e.g. regression coefficient) AND variation (e.g. standard deviation) or associated estimates of uncertainty (e.g. confidence intervals)
☐	☒	For null hypothesis testing, the test statistic (e.g. *F*, *t*, *r*) with confidence intervals, effect sizes, degrees of freedom and *P* value noted<br>*Give P values as exact values whenever suitable.*
☒	☐	For Bayesian analysis, information on the choice of priors and Markov chain Monte Carlo settings
☒	☐	For hierarchical and complex designs, identification of the appropriate level for tests and full reporting of outcomes
☒	☐	Estimates of effect sizes (e.g. Cohen's *d*, Pearson's *r*), indicating how they were calculated

Our web collection on statistics for biologists contains articles on many of the points above.

Software and code

Policy information about availability of computer code

Data collection	The transcription factor binding site prediction was conducted by PlantPAN 3.0 web-tool. Single-guide RNAs of ZmACO2 was obtained from CRISPR-P web-tool.
Data analysis	All the statistical analysis was performed in R v3.5.3 using standard functions. The RNAseq datasets were analyzed by Trimmomatic v.0.36, TopHat v.2.1.1 and Cufflinks v2.2.1. Nucleotide diversity (π), Tajima's D and HKA test were estimated using DnaSP v. 5.0. Association mapping was performed using TASSEL v.3.0.67. Multiple sequence alignment was analyzed by BioEdit v.7.2.5.

For manuscripts utilizing custom algorithms or software that are central to the research but not yet described in published literature, software must be made available to editors and reviewers. We strongly encourage code deposition in a community repository (e.g. GitHub). See the Nature Portfolio guidelines for submitting code & software for further information.

Data

Policy information about availability of data

All manuscripts must include a data availability statement. This statement should provide the following information, where applicable:

- Accession codes, unique identifiers, or web links for publicly available datasets
- A description of any restrictions on data availability
- For clinical datasets or third party data, please ensure that the statement adheres to our policy

Source data are available. The genetic materials that support the findings of this study are available from the corresponding authors upon request. The RNA-seq datasets are available from the National Center for Biotechnology Information, the BioProject and SRA accession numbers are PRJNA705033 and SRR13787135-SRR13787130.

Field-specific reporting

Please select the one below that is the best fit for your research. If you are not sure, read the appropriate sections before making your selection.

☒ Life sciences ☐ Behavioural & social sciences ☐ Ecological, evolutionary & environmental sciences

For a reference copy of the document with all sections, see nature.com/documents/nr-reporting-summary-flat.pdf

Life sciences study design

All studies must disclose on these points even when the disclosure is negative.

Sample size	No sample-size calculations were performed. Sample sizes typically used in the literature for these types of experiments were also used here.
Data exclusions	No data was excluded.
Replication	For all experiments at least three independent biological replicates were performed, and all replicates were included.
Randomization	For all experiments involving phenotyping or tissue collection from plants, segregating populations and a randomized block design were used. The genotypes were planted in a randomly distribution in the field. This ensured that all genotypes were randomized within each experiment.
Blinding	For all experiments involving phenotyping, genotypes were randomized (see randomization). Genotyping was then performed independently of any phenotyping, and the genotype was not known during phenotyping. A randomized block design was used for the phenotyping of families with known genotypes, the distribution of each family in the field was generated randomly before planting. Genotypes and phenotypes were only matched for analysis after all genotyping and phenotyping data had been collected.

Reporting for specific materials, systems and methods

We require information from authors about some types of materials, experimental systems and methods used in many studies. Here, indicate whether each material, system or method listed is relevant to your study. If you are not sure if a list item applies to your research, read the appropriate section before selecting a response.

Materials & experimental systems

n/a	Involved in the study	
☒	☐	Antibodies
☒	☐	Eukaryotic cell lines
☒	☐	Palaeontology and archaeology
☒	☐	Animals and other organisms
☒	☐	Human research participants
☒	☐	Clinical data
☒	☐	Dual use research of concern

Methods

n/a	Involved in the study	
☒	☐	ChIP-seq
☒	☐	Flow cytometry
☒	☐	MRI-based neuroimaging
